# Mach-Zehnder Interferometer Refractive Index Sensor Based on a Plasmonic Channel Waveguide

**DOI:** 10.3390/s17112584

**Published:** 2017-11-09

**Authors:** Da Eun Lee, Young Jin Lee, Eunso Shin, Soon-Hong Kwon

**Affiliations:** Department of Physics, Chung-Ang University, Seoul 156-756, Korea; leedaeun7983@gmail.com (D.E.L.); amitydavil@cau.ac.kr (Y.J.L.); evcraft@cau.ac.kr (E.S.)

**Keywords:** plasmonics, index sensor, waveguide

## Abstract

A Mach-Zehnder interferometer based on a plasmonic channel waveguide is proposed for refractive index sensing. The structure, with a small physical footprint of 20 × 120 μm^2^, achieved a high figure of merit of 294. The cut-off frequency behaviour in the plasmonic channel waveguide resulted in a flat dispersion curve, which induces a 1.8 times larger change of the propagation constant for the given refractive index change compared with previously reported results.

## 1. Introduction

For the problem of designing an enhanced biosensor to detect a tiny variation in a small amount of analyte, it is desirable to achieve a high sensitivity in a small device. Not only is high performance important but it is attractive, from a practical standpoint, to achieve real-time detection, without the use of labelled molecules. Plasmonic refractive index sensors have recently taken centre stage because they allow subwavelength light confinement in the metal surface [1]. In fact, there are two types of plasmonic sensors: the resonant type based on plasmonic resonances and the non-resonant type, based on propagating surface plasmon polaritons [1]. Various plasmonic sensors based on the resonance have been proposed with high sensitivity and compact size [2,3,4,5,6]. However, the figure of merit (FOM = *S*/(linewidth of the resonance)) defined in the resonant refractive index sensor is limited by the broad linewidth resulting from metallic absorption loss in plasmonic sensors [4,7]. Here, sensitivity *S* is defined as *S* = (spectral wavelength shift)/(a given refractive index change). Because the resonant type sensors need to measure the spectral shift of the resonance, the linewidth of the resonant spectrum determines the resolution of the sensor. To avoid such a problem, a non-resonant type of plasmonic sensor was proposed based on the spatial mapping of the refractive index [8]. In this paper, we also propose a non-resonant type index sensor, a Mach-Zehnder interferometer based on a plasmonic channel waveguide.

A Mach-Zehnder interferometer (MZI) is an optical modulator based on the interference between two coherent waves, which travel along different optical paths [9]. In particular, the index change of the material filled in the sensing area of the MZI induces a change of the optical path length, resulting in optical power modulation in the output port. As a result, MZI sensors observe such output power modulation to measure the index change. The sensitivity and the figure of merit, based on the power modulation, are respectively defined by *S* = |d*P*/d*n*|, which is a ratio of the power modulation to the refractive index change and FOM = |[d*P*/d*n*]/*P*|_max_, which is the maximum value of the normalized sensitivity [2,10,11]. The FOM is useful for comparing sensing performance among the index sensors with different experimental set ups. In recent years, several studies on index sensing, based on MZI power modulation, have been reported [10,12,13,14,15,16]. These works clearly show that the FOM of the sensors decrease as one reduces the physical path difference of the MZIs. However, the FOM of the sensor depends not only on the path difference (Δ*L*) but also on the ratio of the wave vector change to small changes in the refractive index, (∂β/∂n). In other words, the FOM for the same path difference can be improved by achieving a high ∂β/∂n by rationally designing the dispersion of the waveguide in the MZI structure. For example, we utilized cut-off frequency behaviour in our study, which is a unique property of a plasmonic channel waveguide with a finite width, to build a certain mode with a high ∂β/∂n.

A plasmonic channel waveguide is a groove-type waveguide with a rectangular cross-section through which surface plasmon polaritons (SPPs) propagate. Recent studies have reported several applications of the plasmonic channel waveguide with a cut-off frequency for optical lasing [17,18], index sensing [4,8] and spectrometers [19]. The cut-off of the SPPs occur in the rectangular grooved channel waveguide with a finite width of the bottom, which is due to confinement of the metallic sidewalls. The cut-off frequency is defined by a certain frequency below which no propagating mode exists. We noticed that the wave vectors of the plasmonic waveguide modes show a drastic change near the cut-off frequency, which can be exploited to develop sensitive refractive index sensors [8].

In this study, we designed a Mach-Zehnder interferometer based on the plasmonic channel waveguide. The new plasmonic MZI can be used for index sensing and simultaneously achieves high sensitivity with a compact sensing area. Obtained by the flat dispersion curve near the cut-off frequency, a high ∂β/∂n contributes to the good FOM of 294, as its maximum. Also, our MZI index sensor takes advantage of the fact that it does not suffer from the resolution limit based on the spectral linewidth. The simple plasmonic channel waveguide structure allows for a compact physical size of 20 × 120 μm^2^. The optical properties of the designed MZI sensor are numerically investigated using the three-dimensional (3D) finite-difference time-domain (FDTD) method.

## 2. Sensing Mechanism

**Figure 1:**
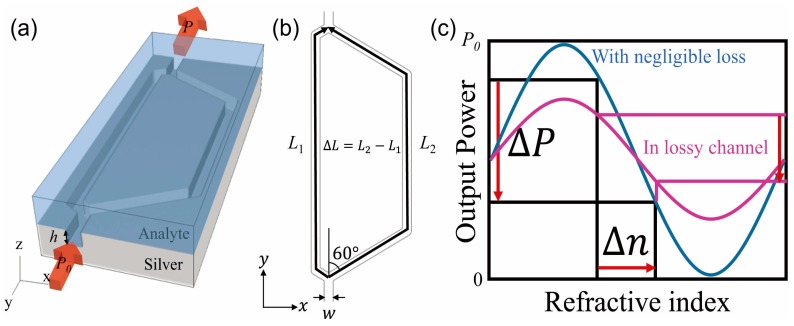
(**a**) A schematic diagram of the proposed MZI index sensor. The channel waveguide on the silver substrate splits into two pathways with different path lengths and combines again at the exit. An analyte with a refractive index of *n* is filled in the waveguide. The input and output power are represented by *P*_0_ and *P*, respectively; (**b**) A top view of the plasmonic channel waveguide MZI. The two waveguides have path lengths of *L*_1_ and *L*_2_ (*L*_2_ > *L*_1_), while having the same width of *w*. The length difference is expressed by Δ*L*. All corner angles are set to 60 degrees; (**c**) a conceptual figure of the output power of MZI modulation as a function of the refractive index of the analyte. Δ*n* denotes the refractive index change and ΔP is the corresponding power modulation.

**Figure 2:**
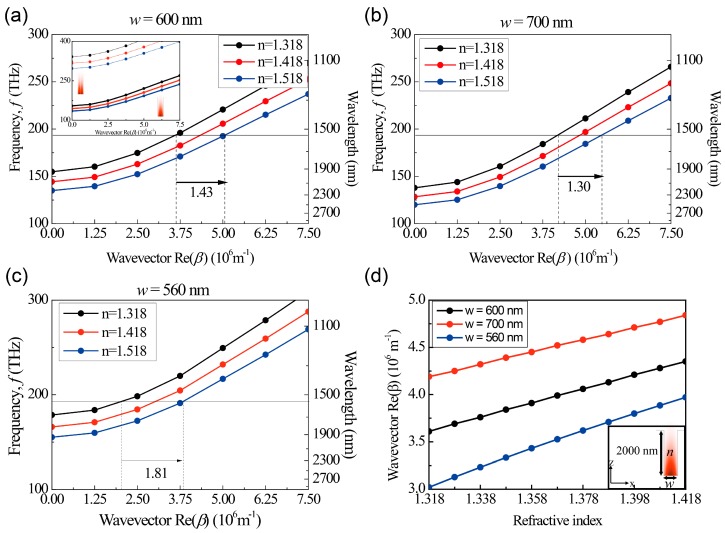
Dispersion curves of the plasmonic channel waveguide, filled with analytes of varying refractive index, for different waveguide widths of (**a**) 600 nm; (**b**) 700 nm; and (**c**) 560 nm; Inset of (**a**) shows the dispersion curves of the higher order mode which has one intensity node at the bottom of the waveguide. The electric field intensity mode profiles in the inset are obtained at the cut-off frequency of each mode. The incident wave is injected with a vacuum wavelength of λ_0_ = 1550 nm. The propagation constant *β* is determined by the dispersion curves, affected by the width w and the analyte’s index *n*; (**d**) Wavevector as a function of the refractive index of analyte for the three different waveguides with the widths of 560 nm (blue), 600 nm (black) and 700 nm (red). The inset shows a mode profile of the z-component of the electric field in the plasmonic channel waveguide with *w* = 600 nm and *n* = 1.318. The waveguide height *h* is set to 2 μm to allow sufficient vertical confinement of light.

The proposed MZI structure consists of two plasmonic channel waveguides with different lengths of *L*_1_ and *L*_2_, which are separated from an entrance waveguide and combined into an exit waveguide, as shown in Figure 1a,b. All the waveguides are designed as the same square groove with a height of *h* and a width of *w* on a silver substrate. The waveguides are filled with an analyte whose refractive index assumed to vary from 1.318 to 1.518. We assumed that the sensing material is filled in the whole region of the MZI in order to avoid difficulties inherent in fabricating a sensing waveguide that is isolated from the reference waveguide. An incident wave with a power of *P*_0_ is injected in the entrance and an output power of *P* is obtained from the exit port. In the simulation, silver is represented by the Drude model with the following Drude parameters: the background dielectric constant *ε_∞_* = 3.1, the plasma frequency *ω_p_* = 1.4 × 10^16^ s^−1^ and the collision frequency γ = 3.1 × 10^13^ s^−1^ [20]. We used a home-made FDTD software. In order to represent infinite free space in the simulations of the MZIs, the uniaxial perfectly matched layer (UPML) was used as the absorbing boundary condition. On the other hand, to calculate the dispersion curves of Figure 2, a periodic boundary condition is used for the y-direction instead of the UPML boundary condition. Considering the skin depth of the silver, tens nanometre, thickness of the silver is set to 300 nm. The height of the analyte is assumed to be 3000 nm which is much larger than the decay length, 900 nm, of the evanescent field of the SPP waveguide mode. In order to excite the SPP waveguide mode, E_z_ linearly polarized light with a centre wavelength of 1550 nm is injected.

A top view of the proposed MZI in the x-y plane is shown in Figure 1b. The curved angles in the two pathways are set equal to 60 degrees. The path length difference of Δ*L* = *L*_2_ − *L*_1_ yields a phase difference between the two output fields, which contributes to power oscillation. In this study, we investigated two path length differences of Δ*L* = 10 μm with *L*_1_ = 112 μm and *L*_2_ = 122 μm and a Δ*L* = 20 μm with *L*_1_ = 122 μm and *L*_2_ = 142 μm. Also, the waveguide width is set as *w* = 600 nm or *w* = 700 nm to investigate the effect of ∂β/∂n. 

Figure 1c shows a conceptual image of power modulation in the plasmonic channel waveguide MZI. The power at the output port (*P*) is determined by the interference between two coherent waves with certain phase differences at the corresponding refractive index. As the refractive index of the analyte changes by an amount of Δ*n*, the following change of the propagation constant will produce different phase differences in the MZI, which causes output power modulation (Δ*P*). Therefore, one can measure the index change Δ*n* by observing the output power modulation Δ*P*. This is the index sensing mechanism in our MZI index sensor. 

The output power of the MZI can be easily calculated from the interference of the two coherent electric fields. Let *E*_0_ be the amplitude of an input wave with the time averaged input power *P*_0_ given by |*E*_0_|^2^/2, then the two split waves have the same amplitude of *E*_0_/2 at the beginning of each path. Because the two waves travel path lengths that differ by Δ*L*, the resulting electric field is a combination of the two fields with a certain phase difference as follows:

(1) $$E=\frac{E_{0}}{2}+\frac{E_{0}}{2}e^{i\beta \Delta L}=\frac{E_{0}}{2}(1+e^{i\beta \Delta L})$$

where *β* is a propagation constant. Thus, the output power *P* becomes

(2) $$P=\frac{{\left|E\right|}^{2}}{2}=\frac{{\left|E_{0}\right|}^{2}}{4}(1+\cos\beta \Delta L)=\frac{P_{0}}{2}(1+\cos\beta \Delta L)$$

where the cosine term is the effect of the interference. It can be seen that the maximum output power becomes the input power and the minimum goes to zero, as depicted by the blue curve in Figure 1c. Because the propagation constant is a function of the refractive index of the analyte, we can expand the propagation constant as

(3) $$\beta (n)\approx \beta_{0}+\frac{\partial \beta }{\partial n}\Delta n$$

Assuming that the variation of the propagation constant is small enough in the small region (Δn) with a refractive index of interest. Here, *β*_0_ is a constant wave vector at a reference point and *n* is the refractive index of the analyte. We can obtain the output power modulation as a function of the change of the refractive index by substituting Equation (3) into Equation (2).

(4) $$P(\Delta n)=\frac{P_{0}}{2}(1+\cos\beta (n)\Delta L)\;\approx \frac{P_{0}}{2}(1+\cos(\beta_{0}\Delta L+\frac{\partial \beta }{\partial n}\Delta L\cdot \Delta n))$$

(5) $$\Delta n_{period}=\frac{2\pi }{\frac{\partial \beta }{\partial n}\Delta L}$$

where the Δ*n*_period_ is the oscillation period of the refractive index, over which a changing index results in one cycle of output power modulation. In Section 4, we will use Equation (5) to confirm that the data achieved by numerical simulations matches our theoretical expectation. On the other hand, the sensitivity, based on the power modulation, is given by

(6) $$S=\left|\frac{\partial P}{\partial n}\right|=\left|\frac{P_{0}}{2}\frac{\partial \beta }{\partial n}\sin\beta \Delta L\right|$$

Also, the figure of merit of the refractive index sensor, based on MZI power modulation, can be calculated from its definition as the following,

(7) $$FOM={\left|\left|\frac{\partial P}{\partial n}\right|/P\right|}_{\max}={\left|\frac{\frac{\partial \beta }{\partial n}\sin\beta \Delta L}{1+\cos\beta \Delta L}\right|}_{\max}$$

One can see that this parameter does not contain the optical power term. Also, it should be noted that the FOM shown in Equation (3) depends both on Δ*L* and ∂β/∂n. Especially, a larger ∂β/∂n results in a larger sensitivity and FOM.

In contrast to the conventional MZI sensors, the plasmonic MZI sensors can have large sensitivity to change in the local refractive index which can be induced by surface molecule binding events because of the strong field enhancement of SPP at the metal/dielectric interface [13]. And the deep-subwavelength propagation of the SPP allows further miniaturization of plasmonic MZI sensors [10,12,13].

In a lossy waveguide, Equation (2) will be modified to a somewhat complex form. We can obtain the modified output power equation, introducing loss factors in the two waves, respectively [13,21]. If we consider the propagation loss, denoted by the attenuation factor *α* and the scattering loss at a corner considered by transmission *T*, then the output electric field changes to

(8) $$E=\frac{E_{0}}{2}{\left(\sqrt{T}\right)}^{2}e^{-\alpha L_{1}/2}+\frac{E_{0}}{2}{\left(\sqrt{T}\right)}^{2}e^{-\alpha L_{2}/2}e^{i\beta \Delta L}$$

Because each path contains two corners, the corner loss occurs twice in the equation. By the same calculation with Equation (2), we can determine the output power, considering waveguide loss, as

(9) $$\begin{array}{l} P=\frac{{\left|E\right|}^{2}}{2}=\frac{{\left|E_{0}\right|}^{2}}{4}\frac{T^{2}}{2}{\left|e^{-\alpha L_{1}/2}+e^{-\alpha L_{2}/2}e^{i\beta \Delta L}\right|}^{2}\\ =\frac{{\left|E_{0}\right|}^{2}}{4}\frac{T^{2}}{2}[e^{-\alpha L_{1}}+e^{-\alpha L_{2}}+e^{-\alpha (L_{1}+L_{2})/2}(e^{i\beta \Delta L}+e^{-i\beta \Delta L})]\\ =\frac{1}{2}\frac{{\left|E_{0}\right|}^{2}}{2}\frac{T^{2}}{2}(e^{-\alpha L_{1}}+e^{-\alpha L_{2}})(1+\frac{2e^{-\alpha (L_{1}+L_{2})/2}}{e^{-\alpha L_{1}}+e^{-\alpha L_{2}}}\cos\beta \Delta L), \end{array}$$

and as a result,

(10) $$\begin{array}{l} P=\frac{P_{0}}{2}W(1+V\cos\beta \Delta L),\\ where\;W=\frac{T^{2}}{2}(e^{-\alpha L_{1}}+e^{-\alpha L_{2}})\;and\;V=\frac{2e^{-\alpha (L_{1}+L_{2})/2}}{e^{-\alpha L_{1}}+e^{-\alpha L_{2}}}. \end{array}$$

Here, *V* is the visibility, defined by (*P_max_* − *P_min_*)/(*P_max_* + *P_min_*). The visibility approaches unity if and only if the amplitude of the two output waves are the same. Because the path lengths over which the two coherent waves propagate are different, such a condition can only be satisfied when there is no loss in the waveguide. Ignoring losses by taking *T* = 1 and *α* = 0, then *W* and *V* both become unity and Equation (2) is retrieved from Equation (10). Using Equation (10), with a non-unity visibility, it can be seen that the output power oscillation does not meet either the input power at its maximum point nor the zero at the minimum point, as illustrated in the purple curve in Figure 1c. This fact will be discussed in the last part of Section 4.

## 3. Dispersion Relation and Wavevector Dependence on the Refractive Index

In order to investigate the change of the propagation constant for analytes with different refractive indices, the dispersion curves of the channel waveguide were calculated. Dispersion curves for three indices, 1.318 (black), 1.418 (red) and 1.518 (blue), are presented in Figure 2a,b. Here, a monochromatic incident light with a wavelength of 1550 nm is injected as an input light source. For the dispersion of *n* = 1.318 and *w* = 600 nm, three important properties can be seen: First, at a wave vector of zero, the frequency of the waveguide mode, defined as the cut-off frequency, has a nonzero value. Below the cut-off frequency, no waveguide mode is allowed. Second, near the cut-off frequency, the dispersion curve is flat. The other five dispersion curves share these general properties, while the values of the cut-off frequencies are modified with changes in the waveguide width *w* and the index of the analyte *n*. Either an increase of *n* or *w* lowers cut-off frequencies in the dispersion curves, as depicted in Figure 2a–c. For example, the cut-off frequencies at *n* = 1.318, 1.418, 1.518 are 179 THz, 166 THz, 155 THz for *w* = 560 nm, 155 THz, 144 THz, 135 THz for *w* = 600 nm and 138 THz, 128 THz, 120 THz for *w* = 700 nm, respectively. In this plasmonic channel waveguide, there can be fundamental and higher order waveguide modes depending on the number of intensity node in the bottom of the waveguide. However, as shown in inset of Figure 2a, since the dispersion curves (dotted lines) of the higher order modes are placed far above the spectral region, telecommunication wavelength, of interest in the fundamental mode, the influences of the higher order modes in sensing can be neglected in the proposed MZIs. In order to obtain the dispersion curve, a periodic boundary condition was used for y-direction and the phase condition at the boundaries was determined by varying the propagation constant. Once the dispersion curves such as Figure 2a–c were obtained for different refractive index, then, the propagation constant corresponding to the target wavelength of 1550 nm was obtained, providing the graph of the propagation constant as a function of the refractive index (Figure 2d).

For the same input wavelength, the narrower channel waveguide has the flatter dispersion curve, which results in a larger change in the propagation constant for an index change of an analyte, as shown in Figure 2a–c. For example, in contrast to the wave vector change of 1.30 × 10^6^ m^−1^ in the waveguide of *w* = 700 nm for an index change from 1.318 to 1.518, the narrower waveguide of *w* = 600 nm has a larger wave vector change of 1.43 × 10^6^ m^−1^ and the further narrower waveguide of *w* = 560 nm has a largest change of 1.81 × 10^6^ m^−1^, as illustrated in Figure 2a–c. Because the dispersion curves of the narrower channel waveguide are flatter near the cut-off point, the wave vector shift (Δ*β*) for the same index change (Δ*n*) becomes larger in the narrower waveguide. This is a unique property of our plasmonic channel waveguide, since common waveguides without a cut-off do not yield such a flat region in dispersion curves.

We checked the optimal height of the channel waveguide in the proposed MZIs. For the channel waveguide with a smaller height, larger scattering loss to free space occurs at the corners. When the height is higher than 1500 nm, the field confinement inside the channel waveguide is strong enough so that the scattering loss becomes negligible. And also the effect of the curved angle was investigated in the proposed MZI. As the angle becomes larger than 90°, the scattering loss in the corner becomes considerably large so that the output powers of MZIs reduces seriously. Therefore, we chose the height of 2000 nm and the curved angle of 60° to minimize the scattering loss effect at the corners in the MZI.

To estimate ∂β/∂n, we examined the wave vector for the three waveguides of *w* = 560 nm, 600 nm and 700 nm as a function of the refractive index. Figure 2d shows that wave vector curves are almost linear with the increasing refractive index of the analyte from 1.318 to 1.418, which is our sensing region of interest. The ratios of the wave vector change to the refractive index change were calculated to be ∂β/∂n = 9.47 × 10^6^ m^−1^, 7.40 × 10^6^ m^−1^ and 6.48 × 10^6^ m^−1^ for *w* = 560 nm, 600 nm and 700 nm, respectively. In other words, the waveguide with a narrower width shows a more sensitive response to the refractive index. The gradients of the two curves were obtained by linear fitting with the linear approximation of the curves under a nearly constant gradient for the small index change. For comparison, the ratios of the wave vector shift to the refractive index change for the other plasmonic structures are estimated as ∂β/∂n~4.10 × 10^6^ m^−1^ for a single silver surface [13], ∂β/∂n~4.10 × 10^6^ m^−1^ for an insulator-silver-insulator with the silver thickness of 100 nm and ∂β/∂n~4.40 × 10^6^ m^−1^ for a silver-insulator-silver structure [10]. Therefore, our structure has a maximally 2.2 times larger ∂β/∂n for the structure with *w* = 560 nm than other plasmonic structures. In contrast to the previously reported plasmonic waveguide structure [10,11,12,13,14], our channel waveguide structure has finite width of the bottom metal surface where SPP is propagating so that there exists a cut-off frequency at zero wave vector. When the target wavelength is close to the flat dispersion curve near the cut-off, large wave vector change can be induced for the change in refractive index, providing 2.2 times larger change of the propagation constant than previously reported results [10,11,12,13,14]. In addition, it is also expected for ∂β/∂n to be enhanced in our structure, as the wave vector point for λ_0_ = 1550 nm and *n* = 1.318 can be placed closer to the cut-off point by careful design of the channel waveguide.

## 4. Output Power Modulation by Refractive Index Change

**Figure 3:**
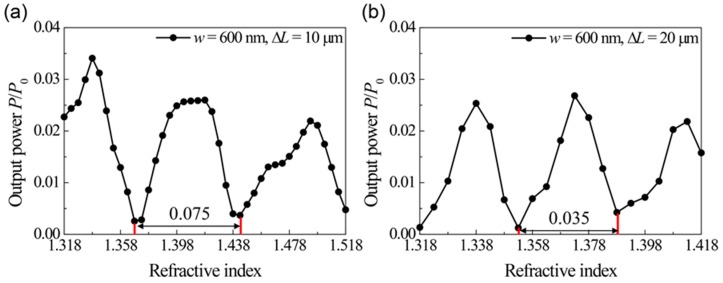

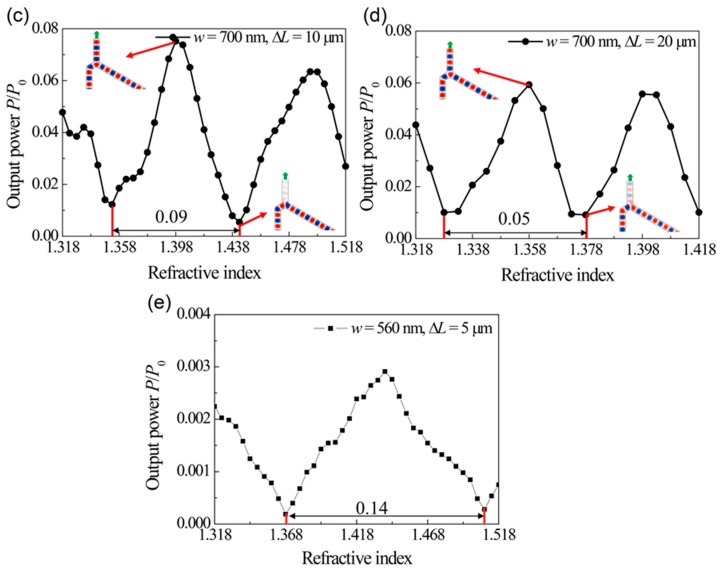
The output power versus refractive index for MZIs with different waveguide widths and optical path length differences. (**a**) *w* = 600 nm, Δ*L* = 10 μm; (**b**) *w* = 600 nm, Δ*L* = 20 μm; (**c**) *w* = 700 nm, Δ*L* = 10 μm; (**d**) *w* = 700 nm, Δ*L* = 20 μm; (**e**) *w* = 560 nm, Δ*L* = 5 μm. Insets show the electric field images near an output port in the x-y plane at the corresponding local maximum and minimum points. Each image is normalized by its maximum amplitude.

To demonstrate index sensing, we investigated the output power modulation versus the refractive index in the plasmonic channel waveguide MZIs. Figure 3a–e show the power oscillation of the normalized output power for the four MZIs with different waveguide widths and path differences. As we discussed before, the oscillation originates from the fact that the propagation constant is a function of the refractive index of the analyte. The obtained values of Δ*n_period_* from Figure 3a–e are presented in the left side of Table 1. The oscillation period (Δ*n_period_*), defined by the index change which induces one cycle of the power modulation, becomes shorter as the path difference (Δ*L*) increases, as expected from Equation (5), which means that a smaller index change can be measured more effectively with larger path differences. In addition, it should be noted that the period also shortened for the narrower waveguide width due to the flatter dispersion curve near the cut-off frequency. In other words, the narrower waveguide MZI is a more sensitive index sensor for the index change, as discussed in Figure 2c. Since the small height of the waveguide and the large curved angle of the MZI induce considerable scattering loss at the corners, we chose the optimal height of 2000 nm and the angle of 60°, in which the scattering loss becomes negligible. And the narrower waveguide width such as 560 nm enlarges the loss in MZIs, as shown in the ten times smaller output power of Figure 3e.

Substituting the values of ∂β/∂n obtained in Figure 2d and Δ*L* into Equation (5), one can calculate the expectation values of Δ*n*_period_ for the given waveguide widths and path differences in Figure 3a–e. For example, taking ∂β/∂n = 7.40 × 10^6^ m^−1^ for *w* = 600 nm and Δ*L* = 10 μm, we get Δ*n*_period_ = 0.085, which is consistent with the simulation result of Δ*n*_period_ = 0.075, as shown in Figure 3a. The expectation values of the oscillation period are estimated from the dispersion curves in Figure 2, as shown in the right side of Table 1. Comparing the Δ*n*_period_ between simulation results and the theoretical expectation from the dispersions, the values almost exactly with a small discrepancy. These discrepancies are because the linearly approximated propagation constant in Equation (3) also has a small nonlinear dependence on the refractive index. Therefore, we can confirm that the oscillation shown in Figure 3 follows Equation (2) and that the ∂β/∂n enhanced by the cut-off mechanism actually determines Δ*n*_period_.

From Equation (5) and Equation (7), the FOM tends to increase as the corresponding oscillation period becomes shorter. In other words, the FOM is better for the narrower waveguide width and larger path difference. One can see the FOMs obtained from Figure 3 in Table 2 are consistent with our expectation. Compared with the previously reported FOM of 177 at Δ*L* = 15 μm [10], our structure with FOM = 123 at Δ*L* = 5 μm, FOM = 215 at Δ*L* = 10 μm and FOM = 294 at Δ*L* = 20 μm has a 2.2, 1.82 and 1.24 times better FOM per unit path length difference (=FOM/ΔL). From these results, one can see that a high ∂β/∂n contributes enhancing the FOM of the sensor. Because smaller group velocity shown in the flatter dispersion curve near the cut-off frequency enhances the absorption and scattering losses, the output power of the MZI with *w* = 560 nm and Δ*L* = 5 μm is ten times smaller than the MZI with *w* = 600 nm and Δ*L* = 10 μm. The MZI with *w* = 560 nm and Δ*L* = 5 μm has a highest FOM per unit path length difference of 24.6/μm, as shown in Table 3, however, small output power can be a drawback for the practical devices. As the waveguide becomes narrower so that the target wavelength is placed close to the dispersion curve near the cut-off, a high FOM per length in the proposed MZI can be achieved, while considerable loss induced by low group velocity is involved in the MZI.

On the other hand, the spectral sensitivity of the proposed MZI, defined by ∂λ/∂n, is a relatively high value of 6500 nm/RIU compared with 2200 nm/RIU at 760 nm of the plasmonic metal-insulator-metal MZI [10] and 4700 nm/RIU at 860 nm [12], 3500 nm/RIU at 700 nm of the plasmonic vertical MZI [13]. The spectral sensitivity can be obtained by the shift of the spectral interference pattern due to the change in the refractive index.

On the other hand, the minimum output powers appearing in Figure 3a–d were obtained as (a) 0.0026, (b) 0.0012, (c) 0.0054 and (d) 0.0091. The maximum powers are (a) 0.0260, (b) 0.0268, (c) 0.0752 and (d) 0.0593. Here, each output power is normalized by the input power. Therefore, the resulting contrast of the output powers are estimated to be (a) 10.0, (b) 22.3, (c) 13.9 and (d) 6.5. As discussed in Figure 2c, the output powers never meet the input power at a maximum point nor are they zero at a minimum point, which is due to the waveguide loss. In our structure, for the corner loss, the transmission *T* is observed as 2% and the propagation loss *α* is 5 × 10^−5^ nm^−1^. The propagation loss includes the metallic absorption loss of the channel waveguide. Considering that the visibility directly affects the FOM of the sensor and because the visibility is maximized when two output powers of the two waveguides are the same, it is desirable to control the two powers to be identical. This work can be done by introducing an additional loss channel or a cut-off filter based on a narrower channel waveguide in only the short path, where the output wave is less attenuated.

Depending on the applications, silver in the proposed MZIs can be replaced by gold. In term of chemical stability, gold is a better choice [2,11,12,14] because of the oxidation layer of the silver with a depth of several nanometres. However, in terms of the metallic absorption loss, silver is better than gold due to silver’s smaller damping constant so that several plasmonic MZI sensors made by silver also have been demonstrated [10,13]. The optical properties of the proposed MZI made by silver, the existence of the cut-off frequency in the dispersion curves, the period of oscillations in output power, etc., were almost maintained in the spectral region near 1550 nm in the MZI made by gold except for that loss is partially enhanced due to the larger metal absorption of gold than silver.

## 5. Conclusions

In our study, a Mach-Zehnder interferometer based on the plasmonic channel waveguide is proposed for index sensing. We calculated the dispersion properties of the plasmonic channel waveguide and demonstrated power modulation versus index change in the MZI structure using the 3D FDTD method. The carefully designed structure has a recorded ∂β/∂n = 7.40 × 10^6^ m^−1^ and Δ*n_period_* = 0.035, which helps to achieve a high figure of merit of 294 for the waveguide width of 600 nm and the path length difference of 20 μm. In other words, the index change of 0.0175 (=Δ*n_period_*/2) can be measured by half a modulation of the output power of the proposed sensor, between the maximum and minimum output power. This structure has a relatively small physical size of 20 × 120 μm^2^ and overcomes the resolution limit based on the spectral linewidth, which is a fundamental limiting factor of LSPR sensors. Also, the introduction of a plasmonic channel waveguide based on the cut-off mechanism results in a large ∂β/∂n, which is beneficial for a very sensitive index sensor. Because the propagation loss in the different pathways causes different powers of two output waves, the minimum power in the output power oscillation appears as 0.0012 rather than zero, with a maximum power of 0.0268, which results in a maximum power contrast of 22.3.

## Figures and Tables

**Table 1:** Oscillation periods of the refractive index in the plasmonic channel waveguide MZI, obtained by FDTD simulation (left) and by calculation from the dispersion curves shown in the Figure 2 (right). The oscillation period indicates the index change, which modulates the output power of the MZI sensor, with one cycle from minimum to minimum. A smaller oscillation period means a more sensitive MZI index sensor. Output power graphs for the MZIs with *w* = 560 nm and Δ*L* = 10 μm or 20 μm are not shown. For the MZI with *w* = 560 nm and Δ*L* = 5 μm, Δ*n_period_* = 0.14 is estimated from the simulation of Figure 3e and Δ*n_period_* = 0.13 is calculated from the dispersion in Figure 2.

Simulation Result	Δ*L* = 10 μm	Δ*L* = 20 μm	Dispersion Expectation	Δ*L* = 10 μm	Δ*L* = 20 μm
*w* = 560 nm	Δ*n*_period_ = 0.060	Δ*n*_period_ = 0.030	*w* = 560 nm	Δ*n*_period_ = 0.067	Δ*n*_period_ = 0.033
*w* = 600 nm	Δ*n*_period_ = 0.075	Δ*n*_period_ = 0.035	*w* = 600 nm	Δ*n*_period_ = 0.085	Δ*n*_period_ = 0.042
*w* = 700 nm	Δ*n*_period_ = 0.090	Δ*n*_period_ = 0.050	*w* = 700 nm	Δ*n*_period_ = 0.096	Δ*n*_period_ = 0.048

**Table 2:** Figure of merit for the four plasmonic channel waveguide MZIs shown in Figure 3.

Figure of Merit	Δ*L* = 10 μm	Δ*L* = 20 μm
*w* = 600 nm	215	294
*w* = 700 nm	142	201

**Table 3:** The path length differences, change of the propagation constant, FOM and FOM per unit path length differences for the typical plasmonic MZIs and the proposed MZI structures.

Reference	Structure	Δ*L* (μm)	∂β/∂n *	FOM	FOM/Δ*L* (μm^−1^)
[10]	Plasmonic MIM-MZI (Ag-SiO_2_-Ag)	15	4.40 × 10^6^ m^−1^	177	11.8
[13]	Single silver surface plasmonic MZI	34	4.10 × 10^6^ m^−1^	122 **	3.59
[14]	Long range Au SPP MZI	1600	4.00 × 10^6^ m^−1^	330	0.21
ours	*w* = 600 nm	10	7.40 × 10^6^ m^−1^	215	21.5
		20	7.40 × 10^6^ m^−1^	294	14.7
	*w* = 560 nm	5	9.47 × 10^6^ m^−1^	123	24.6

* ∂β/∂n was not found explicitly at the references and we calculated the values for the references; ** FOM of [13] was the spectral FOM, defined by the spectral sensitivity (∂λ/∂n) divided by the linewidth of the resonance.
